# Weight loss therapy for clinical management of patients with some atherosclerotic diseases: a randomized clinical trial

**DOI:** 10.1186/s12937-015-0108-y

**Published:** 2015-11-25

**Authors:** Kuat Oshakbayev, Bibazhar Dukenbayeva, Nurzhan Otarbayev, Gulnar Togizbayeva, Nariman Tabynbayev, Meruyert Gazaliyeva, Alisher Idrisov, Pernekul Oshakbayev

**Affiliations:** Department of metabolic syndrome, National Medical Holding, Astana, Kazakhstan; Faculty of pathology and forensic medicine, Medical University Astana, Astana, Kazakhstan; Department of cardiology, National Medical Holding, Astana, Kazakhstan; Faculty of cardiology, Medical University Astana, Astana, Kazakhstan; Department of science and education, National Medical Holding, Astana, Kazakhstan; Faculty of internal medicine, Karaganda State Medical University, Karaganda, Kazakhstan; Faculty of endocrinology, Medical University Astana, Astana, Kazakhstan; Department of science and innovations, Medical University Astana, Astana, Kazakhstan

**Keywords:** Atherosclerosis, Overweight, Weight loss, Endogen metabolic intoxication, Ageing

## Abstract

**Background:**

The prevalence and burden of atherosclerotic (AS) diseases are increasing during the last twenty years. Some studies show a close relationship between overweight and AS, but influence on AS diseases of different weight loss methods are still studying. The purpose of the research was to study the effectiveness of a weight loss program in AS patients in randomized controlled trial, and to develop a conception of evolution of AS.

**Methods:**

A randomized controlled prospective clinical trial including 97 people, from them 71 patients with various AS manifestations. Patients were divided in 2 subgroups for non-drug weight loss program, and conventional drug therapy. The weight loss program included calorie restriction with 100–150 kcal/day, fat-free vegetables, salt diet, and optimum physical activity. Statistical analysis was performed using SPSS for Windows version 17.0.

**Results:**

The weight loss subgroup lost ranging between 7-20 % from an initial weight (*P* = 0.016). Weight loss was achieved due to fatty mass reduction only (*P* = 0.005). Hemoglobin levels (*P* < 0.001), bone mineral density (*P* < 0.001), percentages of water (*P* = 0.006) and muscle masses (*P* = 0.0038) were increased in weight loss subgroup. Ejection fraction (*P* < 0.0001), systolic output (*P* < 0.0001) were increased in patients with coronary artery disease. The weight loss program led to a decrease in symptomatic drugs doses up to total abolition. A conception of AS was developed.

**Conclusions:**

The weight loss program treated the AS diseases; improved laboratory and instrumental parameters, decreased symptomatic drugs doses. AS development is a logical way of ontogenetic ageing of body fat.

**Trial international registration:**

ClinicalTrials.gov NCT01700075.

**Trial national registration:**

State registration is # 0109RK000079, code is O.0475 at the National Center for Scientific and Technical Information of the Republic of Kazakhstan.

## Introduction

The prevalence and disease burden of Atherosclerosis (AS) are increasing. During the last twenty years morbidity and mortality form AS is globally increasing [1]. AS is a disease affecting the blood vessels, arteries of heart, brain and other internal organs [2]. Atherosclerotic fat, atheroma and lipid deposits develops not only in the inner and medial vascular layers, but also it affects the adventitial layer that could be the cause of vasoconstriction worsening the intravascular lumen [3].

Many "Civilization Diseases" such as metabolic syndrome, coronary artery disease (CAD), hypertension, diabetes mellitus (DM), allergic diseases, non-alcoholic fatty liver disease, psoriasis, gout, etc., have similar metabolic disorders such as hyperlipidemia/dyslipidemia, hyperglycemia, hyperinsulinemia, hypercortisolemia, hyperuricemia, microalbuminuria, which can be signs of AS [4, 5]. In this case, the problem of clinical solution AS and its clinical species are socially significant [6]. Pathogenesis of AS is sophisticated integrative process of interaction of endothelial cells of arterial wall, hormonal and metabolic abnormalities, blood components, telomere length, etc. [7].

However, none of them has been universally recognized and proved in practice up to date [8]. Last studies show intimate relationship between obesity/overweight and AS diseases [9]. Increased caloric intake and obesity are recognized to shorten the lifespan [10]. Recent studies indicate that calorie restriction and intense exercise decrease of cardiovascular disease, metabolic disease, oxidative stress, inflammation [11, 12]. Purpose of the research was to study the results of a weight loss program in AS patients in randomized controlled trial. A secondary aim of the study was to develop a conception of evolution of AS.

## Methods and participants

### Study design

Open randomized controlled prospective clinical trial with subsequent development of a conception of evolution of AS based on systematic review of scientific databases.

### Participants

We enrolled a total of 97 adult people of Asian and European ethnicities, from them 71 were patients (34 females) with various clinical manifestation of AS aged 46.5 ± 2.3 years with body mass index (BMI) 29.7 ± 0.8 kg/m^2^ (patient group), and 26 healthy volunteers (12 females) aged 48.5 ± 3.7 years (healthy control group).

The patient group was divided in two subgroups. The weight loss patient subgroup consisted from 31 patients (16 females, aged 42–82 years, mean 47.5 ± 1.9 years, BMI 30.1 ± 1.4 kg/m^2^) with various AS diseases were randomly recruited for the weight loss program (19 Asian, Kazakh and 12 European, Russian). The weight loss patient subgroup included next types of AS diseases: Leriche disease (atherosclerosis of the coxofemoral artery) in 4 patients, CAD with family history more than 10 years was present in 12 patients, CAD with postinfarction cardiosclerosis (PICS) in 6 patients, cerebral stroke in 7 patients, and Alzheimer's disease in 2 patients. All the 31 patients had hypertension, from them 19 patients had DM and the rest 12 patients had an impaired glucose tolerance. All enrolled AS patients had abdominal obesity and before weight loss they took a conventional drug therapy.

The second subgroup for comparison included 40 patients with AS (18 females, aged 26–70 years, mean 45.7 ± 2.2 years, BMI 29.3 ± 1.4 kg/m^2^) who were receiving a conventional drug therapy including hypoglycemic (metformin 500–1500 mg per day, exenatide 5–10 μg per day), lipid lowering (atorvastatin 40 mg per day), antihypertensive (lisinopril 20 mg per day, calcium channel blockers referring to benzodiazepines 90 mg per day), anti-inflammatory (acetylsalicylate acid up to 2 g per day and/or thienopyridine class antiplatelet agent 75 mg per day), and symptomatic therapy.

The study was carried out between October 2009 and April 2012 at Scientific research institute of cardiology and internal diseases (Almaty, the Republic of Kazakhstan) and at Republic scientific center for emergency medicine at National medical holding (Astana, the Republic of Kazakhstan).

Inclusion criteria: written informed consent form for participation in the study; dyslipidemia (blood serum high-density lipoprotein < 1.0 mmol/l, or triglycerides ≥ 1.7 mmol/l or cholesterol ≥ 5.6 mmol/l or both); body fat% > 21; waist circumference in male > 94.0 cm or in female > 80.0 cm; blood pressure (BP) > 140 mmHg of systolic blood pressure (SBP) and > 95 mmHg of diastolic blood pressure (DBP), or ongoing treatment with antihypertensive drugs, fasting glucose > 6.1 mmol/l or treatment with glucose-reducing drugs; absence of contraindications to weight loss; the possibility of treatment for 6 months and dynamic observation for 1 year.

### Outcome measures

The primary efficacy endpoint of the study was the complete recovery from AS diseases. The secondary efficacy endpoint of the study was data imaging methods (doppler-ultrasound, computed tomography scans) and measurement of clinical status presence.

### Randomization

An independent statistician unconnected with clinical practice used computer generated random numbers (SPSS for Windows version 17.0: An IBM Company, Armunk, NY) to prepare randomization lists. The block randomization was two (one on conventional drug therapy, another on weight loss therapy) with stratification by sex, age (47.5 ± 1.9 and 45.7 ± 2.2; *P* = 0.199) and baseline BMI (30.1 ± 1.4 and 29.3 ± 1.4; *P* = 0.34).

### Methods

We diagnosed CAD and PICS in patient according to their case history and electrocardiographic changes of ischemia. We diagnosed hypertension by blood pressure readings and from medical records. Abdominal obesity was assessed waist circumference using the standards for the Asian nationality by the International Diabetes Federation (2005). The weight loss intervention study period was between 2–6 months duration depending on individual patient clinical situation, severity and stages of disease. Physical activity was assessed as the number of steps taken by patients, as determined by the individual pedometer from Hoffmann-La Roche, Ltd (Basel, Switzerland). Mental status was defined by the test of numbers binding by Reitano [13].

We defined anthropometrical indicators including age (years), weight (kg), BMI (kg/m^2^). We also evaluated body composition parameters including as fat mass (in % of total body weight and total kg), visceral fat rating (units), fat free mass (kg), total body water (in % and kg), muscle mass (in % and kg), bone mass (in % and kg), metabolic age (years), basal metabolic rate (kcal per day), and bioimpedance (Ohms) by using Tanita-SC330S Body Composition Analyzer (Tanita Corp., Tokyo, Japan). General clinical study of blood and urine chemistry, liver and kidneys function tests, and imaging methods (GE Vivid 7 Ultrasound; GE Healthcare Worldwide USA, Michigan), bone densitometry (Lunar Achilles Express Ultrasound; GE Healthcare USA, Madison), and computed tomography scans (AG Siemens Somatom Emotion 6, Germany, Muenchen) were performed.

For weight loss we used the weight loss program based on calorie restriction, fat-free vegetables, salt diet (5–6 gr a day), and optimum physical activity [14, 15]. The caloric restriction included 100–150 kcal per day. There was demanded walking with no less of 10,000 steps per day. The exercise model is used for increase of blood circulation and decrease of endogen metabolic intoxication. Doses of previous symptomatic conventional drugs have been competently decreased by the 2–3 day after the treatment start, and came up to full abolition by the 7–10 day as soon as clinical symptoms were improved. A combination of in-person conversations and telephone calls were conducted during the 6-month study period. Weight loss results were assessed by BMI and the Body Composition Analyzer.

### Ethics

Ethical Committee of the Scientific research institute of cardiology and internal diseases (Phone: +7-727-2796751, +7-727-2676851. Email: ncvb@of.kz, ncvb_cardio@of.kz) approved the study. Approval Number is in the protocol #9 from 06.02.2009. Board Affiliation: Health Ministry of the Republic of Kazakhstan.

### Statistics

The two-sample Student’s *t*-test and Odds ratios (ORs) with 95 % Confidence intervals (CIs) were used. The study data were tested on the normal distribution. The study data are presented in Tables as Mean ± Standard Error of the Mean (M ± SE). The correlation analysis (r) and multinomial logistic regression model ORs with CIs were used. P-values of < 0.05 was set as significant. Statistical analysis was performed using SPSS for Windows version 17.0 (SPSS: An IBM Company, Armunk, NY) and Microsoft Excel-2013. Databases of WEB OF SCIENCE, SCOPUS, PUDMED, MEDSCAPE, GOOGLE SCOLAR were used for systematic review analysis of scientific data for working out of development conception of AS.

## Results

We compared the patients and control (healthy) groups concerning metabolic age, basal metabolic rate, anthropometrical data, and body composition (Table 1).

**Table 1:** Anthropometrical data, metabolic data, body composition in the comparing groups

Parameters	Patient group (*n* = 71)		Control group (*n* = 26)		*t*-test	*P*-value
	M	SE	M	SE		
Passport age (years)	46.45	2.29	48.55	3.69	0.484	0.315
Weight (kg)	83.90	2.70	75.61	3.45	1.89	0.039
Height (cm)	167.93	1.12	169.27	1.99	0.587	0.279
BMI (kg/m2)	29.75	0.78	26.22	1.37	2.239	0.014
Fat mass (%)	32.40	1.28	20.27	2.67	4.097	0.00004
Fat mass (kg)	28.34	1.61	17.64	3.18	3.002	0.0017
Visceral fat rating (Unit)	10.04	0.73	6.40	0.70	3.599	0.0003
Fat free mass (kg)	54.80	1.53	58.37	2.04	1.400	0.08
Total body water (kg)	40.91	1.16	41.22	1.63	0.155	0.44
Total body water (%)	48.70	0.80	56.18	2.01	3.458	0.0004
Muscle mass (kg)	52.05	1.46	55.39	1.96	1.367	0.087
Muscle mass (%)	64.19	1.21	75.74	2.56	4.079	0.00005
Bone mass (kg)	3.16	0.07	3.01	0.10	1.229	0.111
Bone mass (%)	3.77	0.09	4.01	0.10	1.784	0.039
Metabolic age (years)	49.00	1.73	42.35	2.60	2.129	0.018
Basal metabolic rate (kcal/day)	1661.6	46.45	1419.8	52.77	3.439	0.0004
Bioimpedance (ohms)	502.10	10.30	467.42	9.11	2.522	0.0067

Abbreviations: BMI, body mass index; M, mean; SE, standard error of the mean

As seen in Table 1, there were no significant differences between the patient group and the control group regarding passport age and height. The patient group had a significantly higher BMI for ≈ 3.5 kg/m^2^ compared with the control group. Fat mass was a significantly higher in the patient group compared with the control group for ≈ 12.1 % or ≈ 10.0 kg. Table 1 clearly makes the inverse relationship between Fat mass percentage and Muscle mass percentage: the greater Fat mass percentage the lower Muscle mass percentage (r = − 066; *P* = 0.0023).

**Fig. 1:**
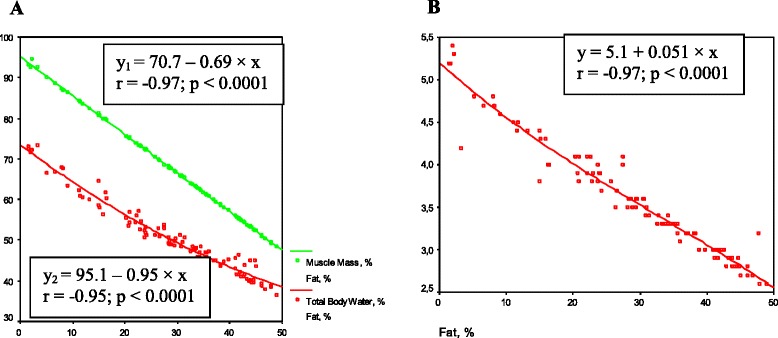
The regression correlation between fat mass (in %) and muscle mass (in %), total body water (in %), and bone mass (in %) in the patient group (*n* = 71). In **a**) x = fat mass (in %), y_1_ = muscle mass (in %), y_2_ = total body water (in %); in **b**) x = fat mass (in %), y = bone mass (in %)

The healthy group displayed significantly greater percentages of Total body water +8 % (*P* = 0.0004), Muscle mass +11.5 % (*P* < 0.0001), and bone mass +0.3 % (*P* = 0.04) than the patient group. The regression linear analysis strongly inverse correlated the relationships between fat mass and muscle/water/bone masses in the patient group (*n* = 71) (Fig. 1).

The patient group also have significantly raised visceral fat rating (t = 3.6; *P* = 0.0003), metabolic age (t = 2.1; *P* = 0.018), basal metabolic rate (t = 3.4; *P* = 0.0004), and bioimpedance (t = 2.5; *P* = 0.0067) than the control group have. Increased body fat mass is associated with accordingly increased bioimpedance of the body.

As seen in Fig. 1, there are significant inverse regression correlations between Fat mass in percent and Muscle, Total body water, Bone masses in percent (*Р* < 0.0001). This data analysis can provide evidence that Fat mass could be the main risk factor for AS patients.

**Fig. 2:**
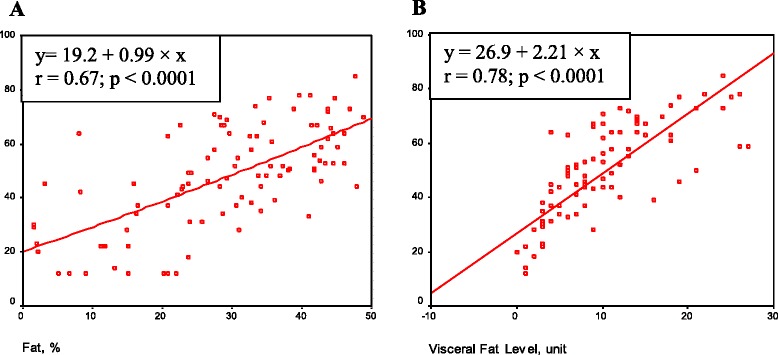
Correlation between metabolic age and fat mass, and visceral fat level in the patient group (*n* = 71). In **a**) x = fat mass (in %), y = metabolic age (years); in **b**) x = visceral fat level (units), y = metabolic age (years)

We studied the regression correlation between the level of obesity (fat mass in % and visceral fat level in units) and the metabolic age in the patient group (*n* = 71) (Fig. 2). As seen here, the increased parameters of obesity (fat mass in %, and visceral fat level in units) significantly correlates with the increased metabolic age (*Р* < 0.0001).

We began the weight loss program in the patient group (*n* = 31) who were randomly assigned into this category. As a result of the treatment average weight lost varied from 6 to 18 kg (or 7-20 %). No differences in weight loss were found between patients with Asian and European ethnicities. The weight loss led to positive changes of the cardiovascular diseases symptoms: SBP and DBP decreased in 94.4 % of patients (*P* < 0.001) for more than 19 % from the initial status (Table 2).

**Table 2:** Blood pressure/hemoglobin/glucose and Lipids profile, and Bone mineral density before/after treatment in the comparative groups

Study groups		SBP, mmHg	DBP, mmHg	Hemoglobin, gram/L	Glucose, mmol/L	Cholesterol, mmol/L	Triglycerol, gram/L	BMD, Units
Conventional patient subgroup, *n* = 40	Before treatment	149.4 ± 3.4	94.8 ± 2.2	132.1 ± 2.2	6.40 ± 0.49	5.60 ± 0.1	2.15 ± 0.05	74.0 ± 2.9
	After treatment	129.6 ± 3.5	93.1 ± 2.1	134.0 ± 2.4	5.27 ± 0.37	5.24 ± 0.16	1.92 ± 0.09	73.2 ± 1.6
Weight loss patient subgroup, *n* = 31	Before treatment	150.1 ± 3.9	99.3 ± 2.9	129.5 ± 2.67	6.42 ± 0.46	5.73 ± 0.13	2.31 ± 0.1	71.6 ± 2.79
	After treatment	121.8 ± 2.1	81.6 ± 1.8	140.3 ± 1.6	4.37 ± 0.38	4.26 ± 0.15	1.62 ± 0.09	97.9 ± 2.8
*P* before and after treatment in Conventional patient subgroup =		<0.001	0.289	0.281	0.036	0.031	0.015	0.405
*P* before and after treatment in Weight loss patient subgroup =		<0.001	<0.001	<0.001	<0.001	<0.001	<0.001	<0.001

Abbreviations: SBP, systolic blood pressure; DBP, diastolic blood pressure; BMD, bone mineral density

Data are presented as Means ± SE

Table 2 shows that in the conventional drug therapy subgroup such parameters as SBP, glucose, cholesterol and triglyceride serum levels were improved significantly (*P* < 0.001). However, the conventional treatment in AS patients did not lead to significant improvement in DBP (*P* = 0.29), Blood hemoglobin levels (*P* = 0.28), and Bone mineral density (*P* = 0.4). Noteworthy, in the weight loss subgroup we observed more significant declines in SBP, DBP, glucose, cholesterol, and triglyceride levels (*P* < 0.001). There were a significant increase in Hemoglobin levels (*P* < 0.001) and Bone mineral density (*P* < 0.001).

The weight loss program led to weight loss ranging between 7-20 % from an initial weight (*P* = 0.016). Importantly, all of the 31 patients weight lost due to reduction of fat mass only (before 26.7 ± 2.9 kg, and after 15.7 ± 2.9 kg, *P* = 0.005; Table 3).

**Table 3:** Parameters in the weight loss patient group before and after treatment (*n* = 31)

Parameters	Before weight loss (*n* = 31)		After weight loss (*n* = 31)		*t*-test	*P*-value
	M	SE	M	SE		
Passport age (years)	47.5	1.9	-	-	-	-
Weight (kg)	89.56	3.59	77.91	3.39	2.359	0.016
BMI (kg/m2)	30.15	1.38	26.23	1.30	2.068	0.021
Fat mass (%)	29.87	2.01	20.23	2.55	2.969	0.0019
Fat mass (kg)	26.75	2.94	15.76	2.98	2.625	0.005
Visceral fat rating (units)	11.73	1.26	8.02	1.64	1.794	0.038
Fat free mass (kg)	62.81	2.18	60.82	2.01	0.671	0.25
Total body water (kg)	43.98	1.69	43.11	1.58	0.376	0.35
Total body water (%)	49.11	1.41	55.33	1.99	2.553	0.0061
Muscle mass (kg)	58.56	2.11	57.55	1.91	0.355	0.36
Muscle mass (%)	65.39	1.87	73.87	2.49	2.723	0.0038
Bone mass (kg)	3.26	0.12	3.05	0.10	1.344	0.091
Bone mass (%)	3.64	0.09	3.91	0.08	2.282	0.012
Metabolic age (years)	56.82	3.89	47.78	3.67	1.669	0.047
Basal metabolic rate (kcal/day)	1837.53	67.42	1495.44	64.01	3.680	0.0002
Bioimpedance (ohms)	505.00	10.22	477.54	8.95	2.021	0.023

Abbreviations: BMI, body mass index; M, mean; SE, standard error of the mean

The data shown in the Table 3 proves that the weight loss in AS patients was due to significant fat loss (*P* = 0.005). The percentages of total body water and muscle masses had also significant tendency to increase in the study endpoint (*P* = 0.006 and = 0.0038, respectively). Lean body mass did not significantly change with fat mass loss (*P* = 0.36).

During the first 2–3 days of the treatment the most of the patients complained of an intense feeling of hunger, slight dizziness, weakness, lower extremity and abdominal muscle tremor, a feeling of warm in the umbilical and/or solar plexus area, and psychogenic fear due to changed eating behavior. All of these uncomfortable feelings were disappearing on subsequent days.

On the 3–5 day after start of the weight loss program the urine of the patients was getting more turbid, muddy and intensively colored (dark) which were not marked before. The urine symptoms were persisted for several days. Microscopy of the urine revealed the turbidity and muddiness were due to the organic salts mainly, such as oxalates, urates, phosphates, and carbonates of calcium and magnesium. An increase in erythrocyte sedimentation rate, leukocyte count in blood samples, and body temperature were observed between 4–10 days after start of the weight loss program.

Regression of AS symptoms were also gradually observed in the weight loss patient subgroup. The patients noticed a physical relief, and an increase in physical and mental workability. Doppler-ultrasound imaging data revealed that blood flow in the lower extremities was restored and the affected gastrocnemius muscle sizes were also restored in all of the 4 patients with the Leriche disease. In CAD patients including with PICS disappeared the angina pectoris (1.52, 95 % CI: 1.24–1.81; *P* = 0.034), improved an exercise tolerance, improved objective electrocardiography indicators of cardiovascular function, increased in ejection fraction from 56.3 ± 1.1 % to 72.1 ± 1.3 % (*P* < 0.0001), and increased in systolic output from 65.4 ± 1.8 ml to 89.6 ± 1.7 ml (*P* < 0.0001). All patients with cerebral stroke and with Alzheimer's disease noticed a decrease in mental fatigue and improvement of memory (1.47, 95 % CI: 1.18–1.77; *P* = 0.039).

As clinical symptoms were improved, as the doses of previous symptomatic drugs were adequately decreased by the 2–3 day after the treatment started. By the 7–10 day after the beginning of the treatment the drugs were abolished. The observational period for the patients was up to 1 year, and there was no recurrence of clinical symptoms of the AS diseases. The weight usually was not regained during the time period. If a person regained overweight again, the clinical symptoms of AS diseases have been gradually manifesting. However, these clinical symptoms were reversed if the weight has been lost again.

## Discussion

Obesity/Overweight is a global public health problem. Overweight is widely acknowledged to be a risk factor for a wide spectrum of cardiovascular, metabolic, neoplastic and musculoskeletal disorders and is estimated to reduce life expectancy by as much as 10 years [16]. The impact of overweight on the risk of AS diseases occurrence evidenced in many of the last cohort studies [17].

One of the first theories of AS development was gerontological, and many researchers consider AS of vessels as one of the determining factors of the ageing process [10, 11]. The "oxidative stress" theory where free radicals can affect intro intima media of arteries could become the cause of one or other undesirable changes in the intima [11, 18]. The inflammatory theory of AS has also place to be that inflammatory process can be cause of lesions of vessel intima [17, 18].

Hyperinsulinemia [19], dyslipidemia, insulin resistance are the way to damage of vascular structures, endothelial function, glucose metabolism with development of AS processes [20, 21]. Intake of excess fats and carbohydrates leads to the overloading of blood transport system. Postprandial hyperlipidemia and long-term storage of fat leads to AS diseases [22, 23]. The hyperlipidemia usually develops after each food intake and lasts for 6 h or more [24, 25]. The body has a limit for storing of fat. If the store is in “overstock” stage, then further hyperlipidemia leads to lipid intolerance.

Bays H. calls the unused adipose tissue as Adiposopathy or "sick fat" which contributes to the emergence of general metabolic disorders and CVD accidents [26]. Adiposopathy can be the main cause of the most cases of adiposity-related metabolic diseases.

Due to “super-nutrient” meal regime a contemporary human begins to accumulate more fat than he is able to use it [27]. Permanent overnutrition changes a balance towards lipid accumulation and deposition. The unused lipids can transform to the body.

The more overweight the more metabolic burden for the body’s organs and systems is [28, 29]. Overweight causes the biological load on the body, and it requires additional thermoregulation, synthetic, trophic, immunological, antitoxic functions, excretion of metabolic products from the tissue [30]. The overweight increases the metabolic burden for the body [31, 32].

Our weight loss program is directed to metabolize of “old lipids” [14, 15]. Weight loss led to significant improvements of cardiovascular symptoms, objective electrocardiography indicators, ejection fraction, systolic output, exercise tolerance and memory, decrease in mental fatigue, etc. Our results are similar to other studies [11, 12]. The weight loss affected fatty mass only whereas lean mass was not significantly changed. One of the problem during weight loss is endogen metabolic intoxication [33]. The exercise model was important part of the weight loss program for cure of endogen metabolic intoxication. During the first few days of the weight loss treatment patients noticed adverse effects related to the symptoms of endogen metabolic intoxication [33]. Strategies related to exercise training improvement the endothelial nitric oxide and prostaglandins pathways [34].

Gender and race can influence for intention to lose weight [35], but our study does not find any differences in weight loss dynamic between Asian (Kazakh) and European (Russian) ethnic groups.

In last decade the importance role of adipose tissue in storing of organic pollutants was considered and highlighted [36]. Adipose tissue can absorb different persistent organic pollutants [37, 38]. Therefore, during weight loss therapy an excretion of organic pollutants from adipose tissue in blood should observe. Consequently, we have developed a method to control an endogen metabolic intoxication during weight loss program.

Hypertension, hyperthermia, insulin resistance, inflammation, allergy can be directed on the maintenance of metabolic rate in the body of patients with overweight/ obesity [39, 40]. Perhaps, the process of atherosclerotic development is a logical output of ontogenetic transformation of lipids [41].

Ability to accumulate of adipose tissue is one of the most important adaptive mechanism for survive. However, nowadays, we are eyewitnesses of another side of the survival mechanism when obesity-related diseases increase steadily [42]. "Since people learned to cook food, they getting hungry twice than nature requires" (B.Franklin, 1706–1790).

**Fig. 3:**
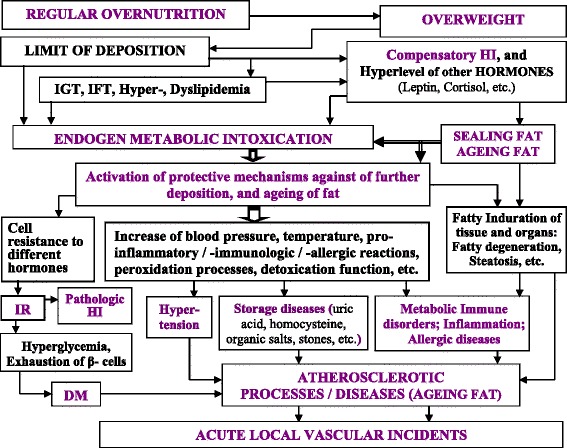
The conception of ontogenetic evolution of Atherosclerosis because of ageing of body fat. Abbreviations: DM, diabetes mellitus; HI, hyperinsulinemia; IFT, Impaired fat tolerance; IGT, Impaired glucose tolerance; IR, insulin resistance

As represented in the Fig. 3 the schematic conception of evolution of AS is related to the ontogenetic ageing of the body fat. The main chain of the suggested conception is: Overnutrition = > Overweight = > Limit of deposition = > Compensatory hyperinsulinemia = > Endogen metabolic intoxication = > Activation of protective mechanisms = > Sealing fat and Ageing fat = > AS processes. Adipose tissue in patients with overweight absorbs of organic metabolic pollutants. Compensatory hyperinsulinemia leads to metabolic abnormality (impaired glucose and fat tolerances, dyslipidemia, etc.). Metabolites overload of the blood transport system and lead to endogen metabolic intoxication. Activation of protective mechanisms (hypertension, hyperthermia, inflammation, allergic, etc.) against of further deposition of fat is images of chronic AS diseases. Long term storing fat in the body leads to ageing of the fat.

### Study limitation

Published studies about possible role of overweight in cause of AS are limited in scope and number. We acknowledge that the randomized clinical trial had a small sample size, and data of comparative or control groups are not completely shown. The clinical results were validated by our usual scientific methods. We should perform the prospective randomized controlled clinical trial with a numbers of patients for more statistical power.

## Conclusions

Thus, the more fat mass is in the body the less muscle, bone, and water masses are. Increased fat mass leads to the increase of bioimpedance and metabolic age. We have developed the weight loss program including a caloric restriction, fat-free vegetables and salt diet with optimum physical activity is effectual treatment method of the AS diseases. The physical activity model was important part for cure of endogen metabolic intoxication of the weight loss program. The program leads to positive change of disease symptoms, improvement in laboratory and instrumental parameters, and competent reduction of previous symptomatic drugs doses up to total abolition. The main conclusion of the study is AS development is a logical output of ontogenetic ageing of body fat. The “ageing lipids” could be used during the weight loss program. The conception of evolution of AS is developed.

## Abbreviations

AS: Atherosclerosis
BMI: Body mass index
CAD: Coronary artery disease
CI: Confidence intervals
DBP: Diastolic blood pressure
DM: Diabetes mellitus
OR: Odds ratios
PICS: Postinfarction cardiosclerosis
SBP: Systolic blood pressure
SE: Standard error of the mean
